# Analysis of influenza transmission in the households of primary and junior high school students during the 2012–13 influenza season in Odate, Japan

**DOI:** 10.1186/s12879-015-1007-8

**Published:** 2015-07-23

**Authors:** Taro Kamigaki, Satoshi Mimura, Yoshihiro Takahashi, Hitoshi Oshitani

**Affiliations:** Department of Virology, Tohoku University Graduate School of Medicine, Sendai, Japan; Division of Pediatrics, Odate Municipal Hospital, Odate, Japan

## Abstract

**Background:**

Households are one of the major settings of influenza transmission in the community and transmission is frequently initiated by school-aged children. We surveyed households with primary school (PS) and/ or junior high school (JH) children for the 2012–13 influenza season in Odate, Japan then characterized the epidemiology of influenza household transmission as well as estimated the serial intervals.

**Methods:**

We delivered a self-reported questionnaire survey to households with PS and/or JH school children in Odate City, Japan. Influenza A (H3N2) virus predominantly circulated during the 2012–13 influenza season. We investigated the epidemiological characteristics of within-household transmission and calculated the serial intervals (SI). SIs were drew by a non-parametric model and compared with parametric models by the Akaike Information Criterion. The covariable contributions were investigated by the accelerated failure model.

**Results:**

Household influenza transmission was identified in 255 out of 363 household respondents. Primary school (PS) children accounted for 45.1 % of primary cases, and disease transmission was most commonly observed between PS children and parents, followed by transmission from PS children to siblings. In primary cases of PS or JH children, younger age and longer absence from school were significantly associated with household transmission events. The mean SI was estimated as 2.8 days (95 % confidence interval 2.6-3.0 days) in the lognormal model. The estimated acceleration factors revealed that while secondary school age and the absence duration > 7 days were associated with shorter and longer SIs, respectively, antiviral prescriptions for primary cases made no contribution.

**Conclusions:**

High frequencies of household transmission from primary school with shorter SI were found. These findings contribute to the development of future mitigation strategies against influenza transmission in Japan.

**Electronic supplementary material:**

The online version of this article (doi:10.1186/s12879-015-1007-8) contains supplementary material, which is available to authorized users.

## Background

Influenza is an acute viral respiratory disease, which is usually self-limiting but can lead to severe complications and reach pandemic proportions. The influenza pandemic of 2009 affected an estimated 24 % of the global population [1] with over 100,000 deaths worldwide [2]. Various non-pharmaceutical measures, such as hand hygiene and school closures, have been implemented, not only during pandemics but also during the periods of seasonal influenza [3]. To optimise these preventive measures, we must understand the dynamics of influenza transmission. Households provide excellent environments for influenza transmission, as contact among household members is exceptionally high [4]. Although school-aged children play an important role in influenza transmission [5], the rate of household transmission is affected by factors such as the structure and the size of the household [6], pre-existing immunity [7] and the household environment. The situation in Japan is rather unique partly because class or grade dismissal is commonly implemented during seasonal influenza epidemics in schools and partly because the proportion of the elderly has reached up to 23 % of the total population in 2010 [8]. Although several studies have investigated the epidemiology of influenza transmission in households during the 2009 H1N1 influenza pandemic [9, 10] and the influenza A(H2N2) of 1957 pandemic [11] in Japan, studies on seasonal influenza have been rarely reported.

Household studies usually measure the secondary infection risk; typically the secondary attack rate (SAR). This proportional parameter enables us to estimate the number of subsequent cases and evaluate the risk ratio, especially by age group. Another important parameter is the serial interval (SI), defined as the time between the onset of specific signs and the symptoms of a primary case and the onset of a secondary case [12, 13]. This parameter characterises the infectiousness profile and determines the rate of epidemic growth. By analysing these variables, we can elucidate (at least partially) the influenza transmission dynamics [14].

We conducted an enhanced influenza surveillance in Odate City, Akita Prefecture, Japan, during the 2011–12 season and found nearly 95 % of patients came to health facilities within 2 days from their onsets [15]. We also conducted a cross-sectional survey among households with primary school (PS) or junior high (JH) school children during that season. This study aimed to characterise the influenza epidemiology in the households of a rural city in Japan and to estimate the SI in this setting.

## Methods

### Study site and design

Odate City is located in Akita Prefecture, northeast of Honshu, Japan. Among its population of 78,946, 15.6 and 31.7 % were registered as under 20 years old and over 64, respectively, in the national 2010 census. A questionnaire survey, designed to obtain information on household influenza transmission, was administered to households with PS or JH children during the 2012–13 influenza season. The questionnaire was handed out to students two times, in January and in March, and collected altogether at the end of March 2013. A total of 5,225 questionnaires were distributed at each time of the survey. Respondents were instructed to record influenza episodes for each household member. The questionnaire requested the demographic information of the influenza cases, students’ grade, the date of fever onset, the status of influenza vaccination, any medical consultation during the clinical course of the disease, duration of school absence and the administration of antiviral drugs. We did not collect the total size of respondents’ households.

### Influenza surveillance during the 2012–2013 season

Influenza surveillance has been undertaken since the 2011–12 season in the city [15]. Throughout the surveillance, an influenza case was suspected if the subject developed fever with cough, runny nose or congested nose. All suspected cases were tested using a commercial rapid test kit. From December 2012 to March 2013, 1,806 suspected influenza cases tested positive, 99.4 % of which were positive for influenza A. The number of PS and JH children cases detected through the surveillance was 352 while the number reported in the survey was 384. The daily numbers of cases reported from the surveillance and the survey were plotted and the correlation was examined by the cross-correlation test. Unfortunately, we could not totally link cases between the surveillance and the survey. In addition, as per a surveillance report of national infectious diseases, 38 out of 40 influenza A strains in Akita Prefecture during that season were influenza A(H3N2); the remaining two were influenza A(H1N1)pdm09 [16]. Only three strains of influenza B were isolated in the prefecture, all belonging to the influenza B Yamagata lineage.

### Data analysis

In the survey, an influenza case was defined as those who reported influenza episodes with a history of medical consultation. A *primary case* is defined as the first influenza case within a household, whereas a *secondary case* is any influenza case following the primary case in the same household. Episodes whose intervals were either zero or more than seven days after the onset of the primary case were excluded from the analysis. Family members were categorised into seven groups: preschool (PreS), PS, JH school, high school (HS), father, mother and grandparents/other adults. The antiviral treatment history, whether subjects had received Oseltamivir, Zanamivir, Laninamivir or Peramivir, was also recorded. Then we characterised the epidemiological factors of primary PS and JH cases between households with and without household transmission events. These variables were the month of onset, the age of primary case, antiviral treatment, pre-season influenza vaccination and duration of school absence of primary cases.

The SI was defined as the interval between the onset of symptoms in a primary and secondary case. Generally SI data constitute a form of time-to-event data and thus it was necessary to consider the censoring time. Unlike in previous studies [13, 17], the questionnaire was distributed through PS or JH children twice in the study season, potentially minimising the data truncation. The SI estimation model was applied from a Hong Kong study [13]. Briefly, the SIs were visualised by a non-parametric model called the Kaplan–Meier estimator, which was compared with the parametric Weibull, gamma and lognormal distributions. Ninety-five percent (95 %) confidence interval (CI) for each model were calculated by using a parametric bootstrap approach with 1,000 resamples. The best-fit parametric model was decided by the Akaike Information Criterion (AIC). Households in which the primary case was a PS or JH student were subjected to multi-variable analysis using an accelerated failure time model to evaluate the contribution of covariates to the SI. The model included the age groups of primary and secondary cases, antiviral prescription, pre-season influenza vaccination and absence duration of primary cases. The transformed regression coefficients derived from the model were interpreted as the acceleration factor (AF). This factor was considered a multiplicative increase (if >1) or decrease (if <1) of the median SI relative to the reference variable.

Categorical data was analysed either by Wilcoxon test or Kurskal-Wallis test and continuous variables were analysed by the chi-square test. The level of statistical significance was determined at p <0.05. All statistical analyses were conducted by R 3.1.0 [18].

### Ethical considerations

All participants were provided with an instruction document stating our research purpose and describing the survey method, along with an informed consent form. Participants completed the questionnaire on agreement and returned that with the written consent. The entire study design was approved by the Ethics Committee of Tohoku University Graduate School of Medicine (ID 2011–268).

## Results

**Fig. 1:**
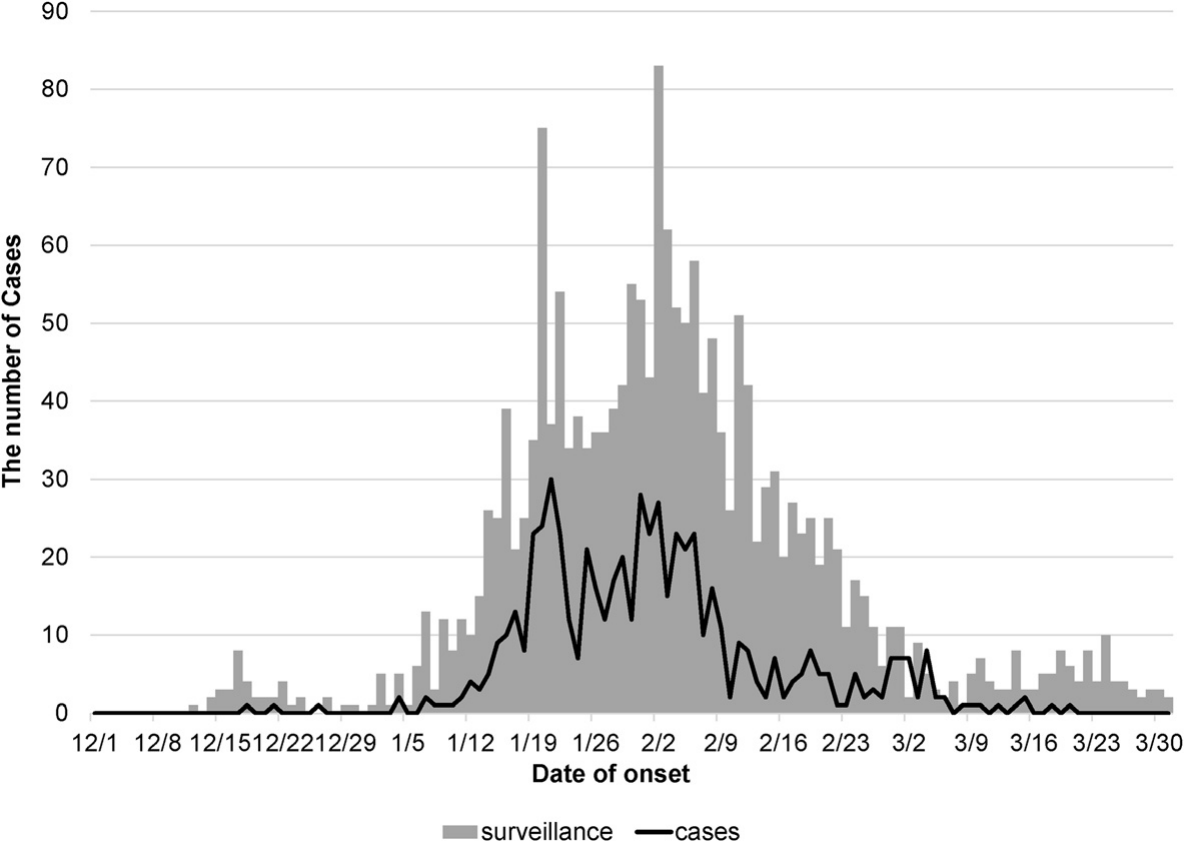
The daily number of influenza cases reported in the questionnaire survey (line) and in a surveillance study of Odate City (grey bars) during the 2012–2013 epidemic season

Among the 2,930 identified eligible households, 363 responded to the survey (12.4 %). Of the responding households, 356 (98.1 %) reported at least one influenza case, and 589 influenza cases were recorded in total. The daily number of surveyed influenza cases was highly correlated with the number of influenza cases detected in an enhanced influenza surveillance of the city [15] (Fig. 1) (correlation coefficient = 0.877). Forty-three percent of the surveyed households reported more than two influenza cases (Table 1). Throughout the study period, 93 households reported two cases and 4 households reported five cases.

**Table 1:** The number and percentages of reported influenza cases among surveyed households

	No. of respondents (%^a^)
Households responding to survey	363
Households with at least one influenza case	356 (98.1)
Households with a single influenza case	202 (56.7)
Households with ≥2 cases	154 (43.3)
2 cases	93 (26.1)
3 cases	47 (13.2)
4 cases	10 (2.8)
5 cases	4 (1.1)

^a^ The percentage for households with at least one influenza case was calculated as the proportion to households responding to survey. Others were calculated as the proportion to households with at least one influenza case

Junior high (JH) and PS students accounted for the majority of cases (71.1 %), but a substantial number of cases developed in mothers, pre-school children and fathers (9.2 %, 6.5 % and 6.1 % respectively). The age distribution of cases reflects their status category (Table 2). Parents, grandparents and other adults accounted for 7.3 % of primary cases and 42.2 % of secondary cases (p < 0.0001). More secondary cases than primary cases were also reported among PreS children.

**Table 2:** The number of both primary and secondary influenza cases by age group, family member category, influenza vaccination, and antiviral drug prescriptions

	No. of total influenza cases (%)	No. of primary cases (%)	No. of secondary cases (%)
Total	589	366	223
Age group^a^			
<5 years	26 (4.4)	8 (2.2)	18 (8.1)
5–9 years	138 (23.5)	101 (27.7)	37 (16.6)
10–19 years	302 (51.4)	228 (62.6)	74 (33.2)
20–49 years	91 (15.5)	23 (6.3)	68 (30.5)
50–64 years	12 (2.0)	1 (0.3)	11 (4.9)
>65 years	18 (3.1)	3 (0.8)	15 (6.7)
Category			
Preschool	38 (6.5)	17 (4.6)	21 (9.4)
Primary school	273 (46.3)	204 (55.7)	69 (30.9)
Junior high school	146 (24.8)	113 (30.9)	33 (14.8)
High school	11 (1.9)	5 (1.4)	6 (2.7)
Father	36 (6.1)	10 (2.7)	26 (11.7)
Mother	54 (9.2)	11 (3.0)	43 (19.3)
Grandparents and other adults	31 (5.3)	6 (1.6)	25 (11.2)
Seasonal influenza vaccination	258 (43.9)	161 (44.2)	93 (43.5)
Antiviral drug prescription	558 (95.1)	364 (94.8)	212 (95.1)

^a^ The total number of primary influenza cases to calculate the percentage by age group was 364

**Fig. 2:**
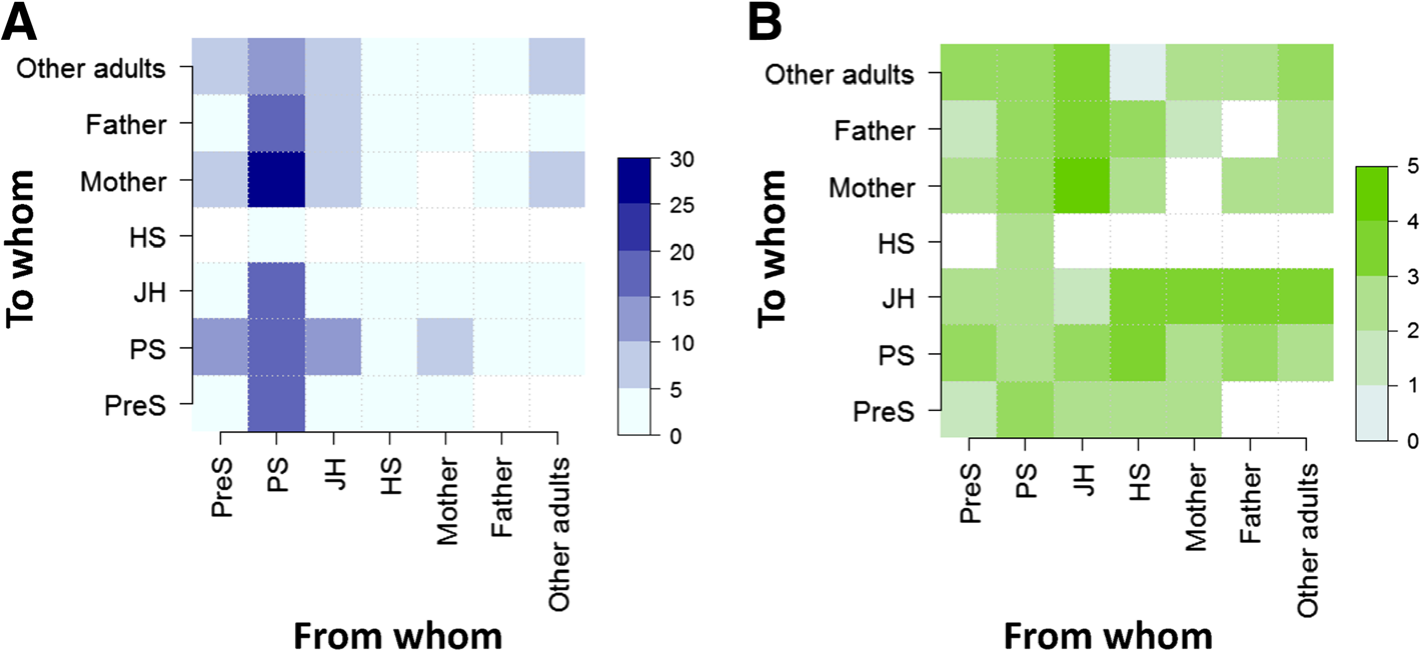
From whom/to whom influenza transmissions in households; (**a**) number distributions, (**b**) distributions of mean onset intervals. Data collected from the Odate questionnaire study during the 2012–2013 season. Abbreviations: PreS, preschool age children, PS, primary school age children, JH, junior high school children, HS, high school children

Among those households reporting influenza cases, 255 reported transmission episodes within the household (Additional file 1: Table S1). Household transmissions from PS, JH and PreS children accounted for 45.1 %, 17.2 % and 13.3 % of all episodes, respectively. Transmissions from PS to mother were most frequently observed (30 episodes), followed by transmissions from PS to father (20 episodes), and from PS to JH or PS to PS (18 episodes) (Fig. 2). The grade distributions were not significantly different between primary and secondary PS cases (p = 0.58), although 38 % of the total episodes developed in grade 1 and grade 2 students. Within households, JH cases were most commonly transmitted to PS children, followed by mothers and grandparents/other adults. Stratified by grades, no significant distribution differences appeared among episodes (p = 0.75), but 60 % of the total episodes developed in JH grade 1 students. Transmissions between siblings, such as PreS to PS and JH to PreS, were also observed. Although the number was small, all of the HS cases were primary cases in household transmissions, except one transmission event from PS to HS. Among parent–parent transmissions, the father was more frequently the primary source than the mother (Fig. 2a, Additional file 1: Table S1).

Table 3 summarises the epidemiological factors observed in primary PS and JH cases between households with and without transmission events. Among the explanatory factors, younger age and longer absence interval in primary cases were significantly associated with household transmission events while the percentages of antiviral drugs prescription as well as influenza vaccination prior to the season were not significantly different.

**Table 3:** The characteristics of epidemiological factors observed in primary cases between with and without household transmission events

	Cases with household influenza transmission (N = 114)	Cases without household influenza transmission (N = 198)	P value
Median onset month	January 2013	January 2013	0.052
Median age of primary cases (range)	11 (6–15)	12 (6–15)	0.01
Antiviral treatment for primary cases (%)	107 (93.9)	189 (95.5)	0.61
Influenza vaccination (%)	90 (45.3)	107 (42.7)	0.79
Median absent days (range)	4.5 (0–10)	4 (0–14)	0.009

Note: Primary cases only included primary school and junior high school children

**Fig. 3:**
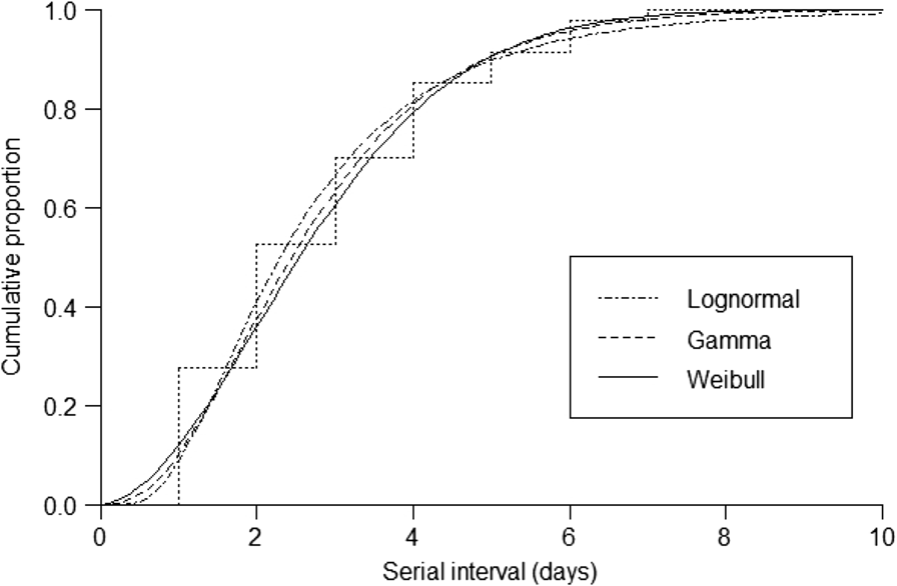
Cumulative distribution of the serial interval of influenza cases fitted by parametric models (lognormal, gamma and Weibull distributions)

The distribution of SIs was constructed for the 298 household transmissions (Fig. 2b) and fitted with parametric models (Fig. 3). These parametric models yielded a mean SI of 2.8 days (95 % CI 2.6-3.0), 3.1 days (95 % CI 2.9-3.3), and 2.8 days (95%CI 2.6-3.0) in Weibull, gamma, and lognormal distributions, respectively. The lowest AIC value was obtained in the lognormal model; hence, this model was used in further analysis. We then performed multi-variable analysis among households whose primary cases were PS or JH students. Specifically, we evaluated the covariate contributions to the SI and the obtained AF using accelerated failure time models. The results are presented in Table 4. Longer SI was more closely associated with JH cases than with PS cases. A significantly shorter SI was associated with secondary cases in both PS and JH children, using fathers as reference. The SI was slightly extended in secondary PreS cases, although the result is not statistically significant. Both antiviral prescription and influenza vaccination shortened the SI in primary cases but not to a statistically significant extent. On the other hand, the duration of absence > 7 days in primary cases was significantly associated with a longer SI.

**Table 4:** Factors affecting the length of serial intervals and the acceleration factors estimated with a multiple parametric model

Variables	No. of cases observed	AF	95 % CI
Primary cases			
Primary school	118	Ref.	
Junior high school	44	1.29	(1.05–1.59)
Secondary cases			
Preschool	22	0.77	(0.55–1.08)
Primary school	31	0.75	(0.55–1.08)
Junior high school	20	0.60	(0.43–0.85)
High school	2	0.71	(0.31–1.66)
Father	26	Ref.	
Mother	39	0.86	(0.64–1.15)
Grandparents and other adults	22	0.93	(0.66–1.30)
Antiviral prescription for primary cases	150	0.81	(0.56–1.18)
Vaccination for primary cases	80	1.06	(0.89–1.27)
Absence interval of primary cases			
≤3 days	42	Ref.	
3 - 7 days	102	1.13	(0.91-1.40)
>7 days	17	1.74	(1.25-2.42)

Note: Households where primary cases were primary shool or junior high school children were included in the analysis

*AF* Acceleration factor,*CI* Confidence interval

## Discussion

Numerous studies have investigated household influenza transmission during periods of seasonal influenza [19, 20] and during the 2009 influenza A(H1N1) pandemic [21–24] and determined that households were a major source of influenza transmission in communities [25, 26]. This study was based on a questionnaire survey administered to households through primary school or junior high school children. From the results, we characterised the household transmission dynamics during the 2012–13 influenza season in Odate City, Japan. During this season, approximately 40 % of responding households reported ≥ 1 member affected by the disease.

Children in pre-school (PreS), primary school (PS), junior high school (JH) and high school (HS) collectively initiated 78 % of household transmissions, of which 80 % were sourced from PS or JH children. We found more household transmission events in younger grades of PS or JH children compared with older counterparts and the regression analysis revealed the odds of household influenza transmission declined with age. One reason is that younger children transmit influenza more efficiently due to closer contacts with other family members [4]. Another reason is that the spread of influenza was initiated in those grades and consequently introduced to the household through those grade groups [27] as the probability of infection in different grades was smaller than that within the same grade [28].

In the present study, secondary cases were most common among the age groups of 10–19 and 20–49 years. Similar trends were reported in other studies. The exception was a U.S. study, in which secondary cases were most common in children aged below 9 years [19]. We determined that 30 and 38 % of cases were transmitted from children to parents and from sibling to sibling, respectively. Intense contact between same age groups and between children and parents has been documented in studies of social mixing patterns [4, 29]. These data suggest that household transmission can be prevented by decreasing the contact intensity through measures such as home quarantine [30]. In addition, longer school absence duration was associated with longer SIs. In Japan, school children with influenza are required by law to stay home until 5 days after the onset and 2 days after the resolution of fever. We did not obtain the timing of symptom resolution; however, the aforementioned association implies that prolonging the time to disease resolution increases the opportunity for virus exposure for a longer time. Carers of infected children need to adopt personal hygiene procedures to minimise disease transmission.

The percentages of reported vaccination as well as antiviral administration of the primary case were not significantly different between households with and without transmission events. Previous studies determined that vaccination exerts no significant effect on secondary infection risk [5, 19]. Similarly, seasonal influenza studies have determined that antiviral treatment imposes no significant reduction in secondary infection risk among household contacts [31, 32]. On the other hand, influenza A(H1N1)pdm09 outbreaks have been significantly [33] or non-significantly [10] reduced by antivirals. As previously reported [15], nearly 95 % of the medical consultations in the Odate population were made within two days of symptom onset; thus, we expected an early antiviral administration could have a protective effect on secondary transmission. However, the extent of protection was insufficient to prevent the transmission. The mean SI of 2.8 days in our study supports a scenario that an exposure to influenza virus is likely to occur prior to antiviral administration. A shorter SI was associated with antiviral administration on a primary case without statistically significant. This suggests the possibility to inhibit the exposure of influenza in relatively late timing by the drug. The infectious period for influenza has not been elucidated, but cohort studies have revealed that viral shedding peaks around the time of symptom appearance [34] and reduces over time [35]. The data of antiviral administration timing can give an opportunity of further analysis of its effect on the SI.

The SI depends on the setting as well as on the causative agent. As per previous studies, the mean SI for influenza A(H3N2) ranges between 1.9 and 3.3 days inclusive [17, 20, 22, 35] and the mean generation time was computed as 3.1 days [36]. Our estimated SI, as well as mean generation time, was comparable to that in studies in Thailand [17] and Hong Kong [22] while another two studies [20, 35] estimated a shorter SI. These differences could be attributed to the right censoring of data [20] or the strict inclusion criteria of secondary contacts among households [35] in the other studies, and to the variation of the sizes of the enrolled households among studies [37]. However, the compatible estimation of SI in our study adds to the evidence that an influenza virus transmission to the next generation occurs within 3 days in household settings.

The AF in secondary cases with fathers as reference indicated that SIs are reduced among children and those shorter SIs were statistically significant among PS and JH children. These findings, which are partially compatible with a previous study [17], suggest that these school age groups can transmit more efficiently because of frequent contact or physical proximity to the primary case in households.

Our study has several limitations. First, since the study questionnaires were distributed through PS and JH students, households without children in these groups were excluded from the analysis. Therefore, we could not investigate the epidemiology of household transmission in general populations. In fact, we conducted an intensive study on households with PS and JH children. Second, we largely lacked information on influenza-free households which consequently led to the low response rate of the survey. Third, we may have underestimated the number of household transmission episodes since some households did not respond to the survey in spite of the instruction. Also, asymptomatic cases can alter the number of secondary influenza episodes in a population; indeed, one study found a substantial proportion of asymptomatic influenza A(H3N2) infections in households [35]. Fourth, we did not obtain the sizes of respondents’ households and thus we could not calculate the SAR as mentioned. Fifth, we relied on households to self-report their medical consultation histories without confirming a laboratory-tested positive influenza result. In Japan, most patients who visit medical facilities with suspected influenza are routinely examined with a commercial rapid test kit [15]. We included the history of medical consultation in our case definition to increase the sensitivity. Sixth, because we collected the questionnaires at the end of March, we missed any influenza cases that occurred after the study period. Finally, we excluded cases whose onsets coincided with the primary onset, potentially missing some transmissions.

## Conclusions

Despite these limitations, we analysed a substantial number of household transmission events in a rural city of Japan and identified school children as the major initial source of infection and estimated a next generation episode within a mean of 2.8 days during influenza A (H3N2) epidemics in the 2012–13 season. Frequent acquirers of familial influenza infection were parents and siblings. A household is considered to provide an opportunity to transmit influenza across different generations. Our findings contribute to the development of future mitigation strategies against influenza transmission in Japan.

## Additional file

Additional file 1: Table S1. Number of household transmission events and mean intervals sorted by from–to transmissions.

## Abbreviations

SAR: Secondary attack rate
SI: Serial intervals
PS: Primary school
JH: Junior high school
PreS: Preschool
HS: High school
AIC: Akaike information criterion
AF: Acceleration factor
