# Supplementary material for: Analysis of influenza transmission in the households of primary and junior high school students during the 2012–13 influenza season in Odate, Japan
Source: BMC Infect Dis. 2015 Jul 23;15:282. doi: 10.1186/s12879-015-1007-8 (PMC4512025; doi:10.1186/s12879-015-1007-8)
Supplement: Additional file 1: Table S1. — Number of household transmission events and mean intervals sorted by from–to transmissions. [file 12879_2015_1007_MOESM1_ESM.docx]

**Supplemental Table1.** Number of household transmission events and mean onset intervals sorted by from–to transmissions.

| Transmissions from | No. of episodes (%) | Mean intervals* |
| --- | --- | --- |
| PS to Mother | 30 (11.8) | 2.6 |
| PS to Father | 20 (7.8) | 3.0 |
| PS to PS | 18 (7.1) | 2.2 |
| PS to JH | 18 (7.1) | 2.5 |
| PS to PreS | 17 (6.7) | 2.6 |
| PreS to PS | 15 (5.9) | 2.7 |
| JH to PS | 13 (5.1) | 2.8 |
| PS to GrandP/Others | 13 (5.1) | 2.8 |
| Mother to PS | 10 (3.9) | 2.4 |
| JH to Mother | 9 (3.5) | 4.2 |
| JH to GrandP/Others | 9 (3.5) | 3.9 |
| PreS to Mother | 8 (3.1) | 1.9 |
| GrandP/Others to GrandP/Others | 7 (1.2) | 3.2 |
| JH to Father | 6 (2.4) | 4.0 |
| GrandP/Others to Mother | 6 (2.4) | 2.5 |
| PreS to GrandP/Others | 6 (2.4) | 3.2 |
| Father to PS | 5 (2.0) | 3.2 |
| JH to PreS | 5 (2.0) | 2.2 |
| GrandP/Others to JH | 4 (1.6) | 3.8 |
| Mother to JH | 4 (1.4) | 3.5 |
| Father to JH | 3 (1.2) | 4 |
| GrandP/Others to PS | 3 (1.2) | 2.3 |
| Mother to GrandP/Others | 3 (1.2) | 2.3 |
| Father to Mother | 2 (0.8) | 2.0 |
| JH to JH | 2 (0.8) | 1.0 |
| HS to JH | 2 (0.8) | 4.0 |
| Mother to PreS | 2 (0.8) | 2.5 |
| PreS to JH | 2 (0.8) | 2.0 |
| PreS to PreS | 2 (0.8) | 1.5 |
| PS to HS | 2 (0.8) | 2.0 |
| GrandP/Others to Father | 2 (0.8) | 2.0 |
| PreS to Father | 1 (0.4) | 1.0 |
| Father to GrandP/Others | 1 (0.4) | 2.0 |
| HS to Father | 1 (0.4) | 2.0 |
| HS to Mother | 1 (0.4) | 3.0 |
| HS to PreS | 1 (0.4) | 2.0 |
| HS to PS | 1 (0.4) | 4.0 |
| Mother to Father | 1 (0.3) | 1.0 |

*Unit = days
